# Viroids of the Mediterranean Basin

**DOI:** 10.3390/v16040612

**Published:** 2024-04-15

**Authors:** Maria Kaponi, Panayota E. Kyriakopoulou, Ahmed Hadidi

**Affiliations:** 1 Plant Virology Laboratory, Benaki Phytopathological Institute, Stefanou Delta 8, Kifissia, 14561 Athens, Greece; 2 Department of Crop Science, Agricultural University of Athens, Iera Odos 75, 10447 Athens, Greece; pek.chryssohori@gmail.com; 3 United States Department of Agriculture, Agricultural Research Service, Beltsville, MD 20705, USA; ahadidi@yahoo.com

**Keywords:** Mediterranean basin, viroids, viroid diseases, host range, incidence in each Mediterranean country

## Abstract

There has been substantial progress in the Mediterranean countries regarding research on viroids. Twenty-nine viroid species, all belonging to *Pospiviroidae* and *Avsunviroidae* genera, have been detected in the Mediterranean Basin. Not only have detection methods, such as reverse transcription-quantitative polymerase chain reaction and next-generation sequencing, been used for viroid detection, along with molecular hybridization techniques allowing for rapid detection, identification, and characterization of known and novel viroids in these countries, but eradication measures have also been taken that allowed for the efficient elimination of certain viroids in a number of Mediterranean countries. The eradication measures were followed as recommended by the European and Mediterranean Plant Protection Organization, which is known by its abbreviation, EPPO. The Mediterranean Region has been a niche for viroids since ancient times due to the warm climate and the socio-cultural conditions that facilitate viroid transmission among different host plant species.

## 1. Introduction

**Figure 1:**
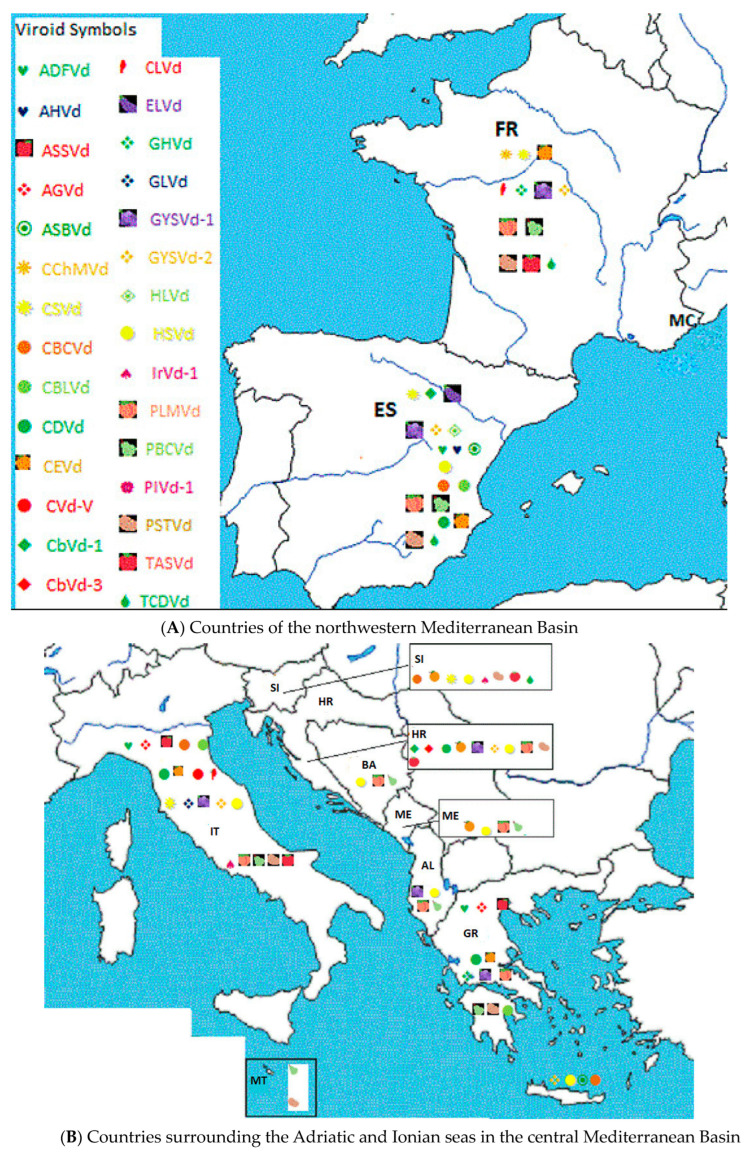

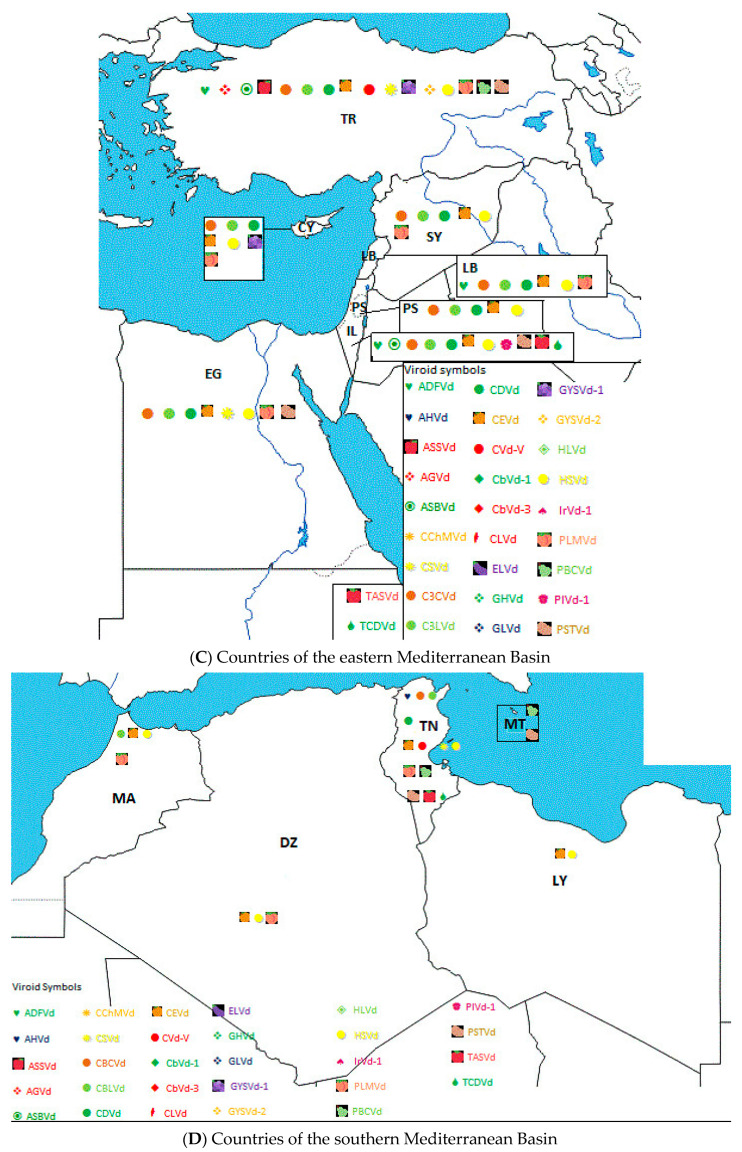
Viroids reported in countries of the Mediterranean Basin. Alphabetic list of countries with their ISO codes: (AL) Albania, (DZ) Algeria, (BA) Bosnia and Herzegovina, (HR) Croatia, (CY) Cyprus, (EG) Egypt, (FR) France, (GR) Greece, (IL) Israel, (IT) Italy, (LB) Lebanon, (LY) Libya, (MT) Malta, (MC) Monaco, (ME) Montenegro, (MA) Morocco, (PS) The Palestine Authority, (SI) Slovenia, (ES) Spain, (SY) Syria, (TN) Tunisia, and (TR) Turkey.

The Mediterranean Basin (also known as the Mediterranean Region) is the region of land around the Mediterranean Sea with a coastline of about 46,000 km (about 28,600 miles). The Mediterranean Basin covers portions of three continents, north Africa, southwest Asia, and south Europe, that include 22 countries with a population of about 500 million people. Figure 1 shows viroids reported in countries of the Mediterranean Basin.

### Viroid Acronyms

ADFVd, apple dimple fruit viroid; AHVd, apple hammerhead viroid; ASSVd, apple scar skin viroid; AGVd, Australian grapevine viroid; ASBVd, avocado sunblotch viroid; CChMVd, chrysanthemum chlorotic mottle viroid; CSVd, chrysanthemum stunt viroid; CBCVd, citrus bark cracking viroid; CBLVd; citrus bent leaf viroid; CDVd, citrus dwarfing viroid; CEVd, citrus exocortis viroid; CVd-V, citrus viroid V; CbVd-1, coleus blumei viroid 1; CbVd-3, coleus blumei viroid 3; CLVd, columnea latent viroid; ELVd, eggplant latent viroid; GHVd, grapevine hammerhead viroid; GLVd, grapevine latent viroid; GYSVd 1, grapevine yellow speckle viroid-1; GYSVd 2, grapevine yellow speckle viroid-2; HLVd, hop latent viroid; HSVd, hop stunt viroid; IrVd-1, Iresine viroid 1; PLMVd, peach latent mosaic viroid; PBCVd, pear blister canker viroid; PlVd-1, plum viroid 1; PSTVd, potato spindle tuber viroid; TASVd, tomato apical stunt viroid; TCDVd, tomato chlorotic dwarf viroid.

List of symbols corresponding to viroids and viroid acronyms:


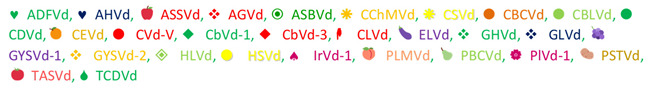


The Mediterranean Region has been the scene of intense human activity for millennia, including the development of many ancient civilizations as well as agriculture. Fruit and nut trees, grapevine, vegetable and field crops, ornamental crops, and other crops are widely cultivated in the region.

In the Mediterranean Region, natural viroid infection involving members of the families *Pospiviroidae* and *Avsunviroidae* occurs widely. The genera in these families include *Pospiviroid*, *Hostuviroid*, *Apscaviroid*, *Cocadviroid*, and *Coleviroid* (*Pospiviroidae*); and *Avsunviroid*, *Pelamoviroid*, and *Elaviroid* (*Avsunviroidae*). Currently, there are 44 viroid species recognized by the International Committee of Taxonomy of Viruses (ICTV) [1]. The ICTV requires at least two independent criteria for establishing new viroid species, a sequence identity lower than 90% over the entire viroid genome and the existence of some divergent biological properties between different species [2].

The Mediterranean climate is characterized by mild wet winters and warm to hot, dry summers. That climate most likely enhances the expression of viroid disease symptoms on infected plants. Natural viroid infection has been reported on potato, tomato, cucumber, pepper, eggplant, hop, peach, apple, pear, citrus, avocado, and grapevine. Viroid-infected ornamental plants such as certain chrysanthemum show viroid symptoms, whereas other ornamentals such as garden dahlia and spurflower may be symptomless. Currently, only one cereal grain crop, sorghum, has been reported from Turkey to be infected with peach latent mosaic viroid [3].

Coconut cadang-cadang viroid and coconut tinangaja viroid are known to infect coconut palm in the Philippines and oil palm in south east Asia and cause significant coconut yield reduction [4,5]. However, no viroid infections were reported on date palm in the Mediterranean Region. This article summarizes the incidence of viroids and viroid diseases in the Mediterranean Region.

## 2. Geographical Distribution and Host Range of Viroids in the Mediterranean Basin

Twenty-nine viroids were identified and characterized in the Mediterranean Basin. Table 1 shows the geographical distribution and host range of these viroids.

## 3. Viroids of Woody Plants: Fruit and Nut Trees, and Grapevine

So far, viroids that have been reported in fruit trees (pome, stone, citrus, fig, mulberry, avocado, mango, pomegranate), nut trees (walnut, almond, pistachio),and grapevine in the Mediterranean Basin belong to six viroid genera: *Pelamoviroid* (AHVd, PLMVd), *Avsunviroid* (ASBVd), *Hostuviroid* (HSVd), *Apscaviroid* (ADFVd, ASSVd, AGVd, CBLVd, CDVd, CVd-V, GLVd, GYSVd-1, GYSVd-2, PBCVd), *Cocadviroid* (CBCVd), and *Pospiviroid* (CEVd, PSTVd).

The apscaviroids are prevalent in pome fruits, whereas two viroids from the other genera, namely, HSVd and PLMVd, are prevalent in stone fruit trees in several Mediterranean countries. PLMVd has been found frequently in peach and occasionally, in plum, sweet cherry, and apricot germplasm in very significant percentages [57]. HSVd has been detected also frequently in several stone fruit species, like apricot, peach, plum, nuts like almond, other fruit trees like fig, pomegranate, and mulberry, as well as in grapevine [4,57,76,83,89,92,93,94,96,99].

A new viroid has been identified in grapevine by next-generation sequencing (NGS) [141], in addition to four different viroids since 1985, besides CEVd and HSVd. In grapevines, although viroids usually do not cause severe symptoms, some of them are disease agents in certain environmental conditions or in combination with certain viruses, and some others, like HSVd or CEVd, may cause severe diseases in other crops. 

**Figure 2:**
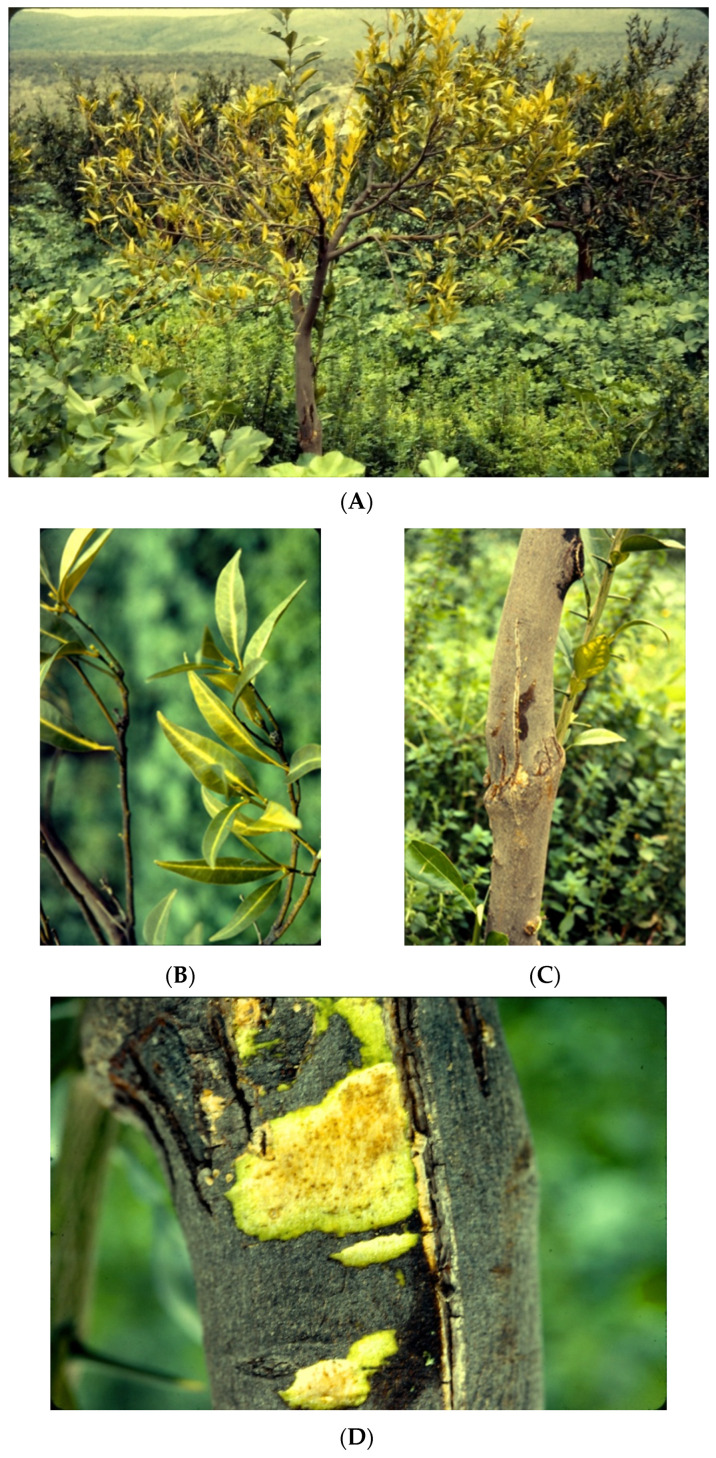
A mandarin tree from Greece with cachexia (xyloporosis) symptoms. (**A**) The whole tree, (**B**) Shoots and leaves with perineural chlorosis, (**C**) Outside the tree trunk, (**D**) Detail of inside the tree trunk after removing the phloem.

HSVd is the viroid with the widest host range. Despite its infection being usually latent in stone fruits, it has been associated with serious disorders of economic importance; the first is the dapple fruit disease of plum and peach, “kirin-ka” [142], and the second, an apricot fruit disorder known as “fruit degeneration” characterized by fruit rugosity and the loss of organoleptic properties [143]. On the other hand, in citrus fruit trees, CEVd and HSVd have been reported in most Mediterranean countries and are among the most prevalent citrus viroids in the region [144], because the usual citrus rootstock (sour orange) is tolerant to diseases caused by them, namely, citrus cachexia (xyloporosis) (Figure 2) and citrus gummy bark (CGB), which are caused by variants of HSVd [81], and citrus exocortis, caused by the homonymous *Pospiviroid*. These diseases pose a threat to the used citrus tristeza virus (CTV)-tolerant trifoliate rootstocks as they are susceptible to them.

Reliable detection and identification methods such as molecular hybridization, RT-PCR, and NGS [4,5] were developed to allow for extensive research and surveys for viroids. Molecular hybridization and RT-PCR have been widely used in surveys to identify CEVd and HSVd in many different Mediterranean countries (Figure 1, Table 1).

## 4. Viroids of Herbaceous and Ornamental Crops

Viroids infecting herbaceous and ornamental crops in the Mediterranean Basin belong to six viroid genera: *Pelamoviroid* (CChMVd, PLMVd), *Elaviroid* (ELVd), *Hostuviroid* (HSVd), *Cocadviroid* (HLVd), *Coleviroid* (CbVd 1, and 3), and *Pospiviroid* (PSTVd, CEVd, CSVd, CLVd, IrVd-1, TASVd, TCDVd) [2,145,146,147]. These viroids, with the exception of HLVd, have been reported in vegetables (tomato, potato, pepper, eggplant, carrot, broad bean, chickpea), oil crops (rapeseed), several ornamental crops like chrysanthemum, marguerite daisy, common ragwort, cape gooseberry), spurflower, common periwinkle, petunia, trailing petunia, and climbing nightshade, as well as in sorghum, the latter being the first report of a viroid infecting poaceous monocots in the Mediterranean and worldwide [3].

In hop, it is infected by HLVd (causes hop latent), CBCVd (causes severe hop stunt), and HSVd (causes hop stunt).

PSTVd has quarantine and certification status in the European Union (EU); however, it has been detected in several ornamental cape gooseberry, common ragwort, petunia, night blooming jessamine, trailing petunia, climbing nightshade, angel trumpets, herbaceous crop seeds (tomato, pepper, eggplant), and tubers of potato in some Mediterranean countries and in the rest of Europe.

## 5. Viroids Not Reported in the Mediterranean Basin

Other known viroids have not been identified in the Mediterranean Basin. They include apple chlorotic fruit spot viroid, apple fruit crinkle viroid, citrus viroid VI, coconut cadang-cadang viroid, coconut tinangaja viroid, coleus blumei viroid 5, coleus blumei viroid 6, coleus blumei viroid 7, pepper chat fruit viroid, portulaca latent viroid, persimmon viroid-2, and tomato planta macho viroid.

## 6. Incidence of Viroids and Viroid Diseases in Countries of the Mediterranean Basin

### 6.1. Albania

There are a few occurrences of HSVd in apricot (30%), fewer in plum (7–30%), in peach (6.7–7%) and in grapevine germplasm [57,76,148,149]. On the other hand, while the incidence of PLMVd in Albanian peach is high, 52%, it is low (3.3%) in plum and in apricot [148,149]. PBCVd is present in pear; four native pear cultivars tested positive for PBCVd at an incidence rate of about 13%, 7/55 trees [119].

### 6.2. Algeria

PLMVd was reported at infection rates of 17.6% in apricot and 5.4% in peach [57,149], while the presence of HSVd was sometimes simultaneous with that of PLMVd [149]. Rouag et al. [150] reported that by using tissue print hybridization (TPH) assays, they found 28 of 531 stone fruit samples (5.2%) positive for PLMVd and HSVd; both viroids were present in imported peach and apricot varieties (14% and 5.8% infection, respectively), but not in cherry, almond, plum, and myrobalan. The prevalence of CEVd in citrus has been studied by real time RT-PCR. A total of 50 citrus samples were collected from symptomatic CEVd-infected plants in Mitidja (North Algeria), and CEVd was detected in 38 of them, estimating the prevalence of CEVd at 76% [148,151].

### 6.3. Bosnia and Herzegovina

HSVd was detected in three apricot and two plum trees in the northern part of the country. PLMVd was detected in 39 peach trees, widely distributed throughout the country and during the whole year. Both native and imported cultivars of *Prunus* species were found infected by these two viroids [87,148]. Also, PBCVd was detected in 13% of pear samples tested by TPH assay. The assay gave positive signals for PBCVd which confirmed dot-blot and Northern-blot hybridization assays. PBCVd infected pear trees of at least 10 different cultivars [120].

### 6.4. Croatia

The incidence of PLMVd was estimated at 94% in nectarine and 42% in peach [104]. In addition, highly pathogenic variants of CEVd and milder variants of HSVd and CDVd were found to co-infect citrus orchards in the Croatian coast and islands during molecular analyses. This finding, along with other analyses which revealed the existence of pathotypes not believed to be endemically present in the Mediterranean Basin confirmed that CEVd, and possibly other citrus viroids, were introduced into the country with imported plant material [47,148,152].

PSTVd was first detected in 2009 on symptomless ornamental plants in the country. All infected plants were destroyed. Then, PSTVd was detected, during a survey carried out from 2009 to 2012 on pospiviroids in ornamental plants, in eight plants of climbing nightshade and Paraguay nightshade which were then destroyed. In addition, one climbing nightshade plant was found infected by TASVd by one-step RT-PCR, followed by mechanical inoculation on tomato plants to observe symptom expression [133,153,154].

GYSVd-1, GYSVd-2, and HSVd were detected in vineyards. They were found by NGS of total RNA samples from four Croatian autochthonous grapevine varieties [75,155]. CbV-1 and CbVd-3 were reported in asymptomatic spurflower. CbVd-1 was found in one spurflower cultivar in a single infection and in another cultivar in a mixed infection with CbVd-3, while the latter was found in a third spurflower cultivar in a single infection [65,66].

### 6.5. Cyprus

The HSVd incidence in apricot was estimated at 10.4% and in peach at 7.5% [57,89,149]. In citrus, both CEVd and HSVd are widespread, and there has been an effort to eliminate them from the original maternal material of local varieties using in vitro shoot-tip grafting [156,157]. Citrus accessions were produced by micrografting. Cypriot lemon ‘Polýphori’ and ‘Lapithou’,‘ Arakapas’ mandarin, ‘Jaffa’ and ‘Siekeriko’ orange, Bergamot, ‘Frappa’, and ‘Coumantantas’ were found viroid-free and citrus psorosis virus (CPsV)-free. Testing for viroids and CPsVwas being continued for all plants which were produced by in vitro shoot-tip grafting until 2011 [158].

During a survey for CEVd in commercial groves, representing 25 species and varieties of the main citrus-producing areas of Cyprus, 88.3% (506 of the 573 trees sampled) were found CEVd-infected when indexed by grafting to ‘Etrog’ citron seedlings. Levels of infection by CEVd were lower in newer imports than those made before the 1940s. In greenhouse tests, CEVd was readily transmitted from infected citron to healthy citron and gynura by knife-blade inoculation. Re-transmission of CEVd from infected gynura back to citron was successful, fulfilling Koch’s postulates [55]. In another survey, CEVd was the most widespread viroid (incidence 80.5%), while HSVd was detected in 53.3% of the 500 samples tested. CBLVd, CDVd, and CBCVd were also identified at lower rates of 12.2%, 9.6%, and 7.7%, respectively. CEVd+HSVd were the most common viroid combination (69.3%), while CEVd + CBLVd, CEVd + CDVd, and CEVd + CBCVd were detected at 9.7%, 9.5%, and 9.4% of the double viroid mixtures (25.4%), respectively. Triple viroid combinations were also recorded at 9.8% of the tested samples with CEVd and HSVd identified in all mixtures. CVd-V was not detected in any of the samples. Only 7.5% of the samples tested were negative to viroid infection, indicating the need for dissemination of virus/viroid-free propagating material [56,148,158,159,160,161]. In this country, a citrus certification scheme had been lacking 30 years ago, and most of all, there is widespread use of the sour orange rootstock, which is tolerant to viroids, despite being susceptible to CTV. Sour orange is still used on the island as the main rootstock due to its excellent adaptation to the local environmental conditions [55]. Viroids pose a threat to the citrus industry, as most of the commercial citrus rootstocks that are resistant/tolerant to CTV seem to be highly susceptible to viroid infection. Papayiannis [56] developed an RT-qPCR method, allowing detection of CEVd and HSVd, that was 1000-fold more sensitive compared to conventional RT-PCR.

GYSVd-1 and HSVd (very often in association) were also detected in grapevine germplasm [76]. Moreover, the incidence of PLMVd in peach samples was estimated at 12.5% [148,149].

During the years 2010–2012, extensive surveys were conducted by the Cypriot Ministry of Agriculture for determining the presence or absence of the pospiviroids: PSTVd, TASVd, TCDVd, and CSVd. In total, 400 samples from ornamental species, like chrysanthemum and jasmine, as well as from vegetable crops such as potato, tomato, eggplant, cucumber, zucchini, squash, and melon were subjected to the molecular tests defined by EPPO. The results confirmed the absence of these viroids from Cyprus [157].

### 6.6. Egypt

CBLVd and CDVd were detected in citrus budlings and in volkamer lemon originating in the country [37,38,148,159,160,161]. In 2004, Torres et al. [149] estimated the infection of HSVd in apricot samples was 57%, in plum, 6.6%, and in peach, 3.3%. Later, the infection rate of HSVd in peach and pear samples was estimated at about 93% [83]. In a following study, HSVd infection in several fruit species was about 2% in apple, 6% in plum, 7% in apricot, 10% in mandarin, 16% in mango, 25% in sweet orange, 40 % in pear, and 65% in peach [52]; in grapevine, the infection rate was estimated from 8% to 12% [52,84]. Citrus gummy bark disease was shown to be induced by a molecularly characterized variant of HSVd [85,86].

The incidence of PLMVd infection in fruit trees was about 45% in peach, 35% in plum, 23% in pear, 18% in mango, 5% in apple, and 2% in apricot [52]. PSTVd was detected in potato [129,145] at the low incidence of 2.5% [129,153,154]. CSVd in chrysanthemum was also detected [34,148]. The infection rate of CEVd was about 15% in sweet orange, 4–12% in grapevine, and 1% in mango [52,84].

### 6.7. France

CSVd, TASVd, and PSTVd have been eradicated from this country [148,153]. The frequency of PLMVd is relatively low (about 15%), and its distribution is restricted [148,162,163]. PBCVd is among the first viroids found in France [121,122,162,163].

History of CSVd eradication in France: CSVd is classified as a quarantine pest in chrysanthemum in the EU. A report in 2010 in a nursery in southwest France of stunted marguerite daisy plantlets with yellow deformed leaves and terminal necrosis, symptoms resembling those reported for CSVd in marguerite daisy, led to RT-PCR tests on five symptomatic plants. Thirty-five symptomless mother plants of marguerite daisy cv. Butterfly were tested by RT-PCR, and all were shown to be CSVd-positive. Estimation of the extent of CSVd contamination in marguerite daisy, by sampling 11 other cultivars originating from other nurseries, showed that besides Butterfly, five other cultivars out of 11 were found CSVd-positive in the absence of clear symptomatology [164]. The finding of its significant prevalence in French marguerite daisy cultivars, frequently in the absence of clear symptomatology, raised the possibility that infected marguerite daisy might become a reservoir for future chrysanthemum infection; therefore, eradication measures were taken, and eradication of CSVd in France was reported in 2023 [148], while back in 2012, it was still present, with restricted distribution in French marguerite daisy [165].

HSVd was detected both in mainland and in the island of Corsica [148]. TCDVd was reported in a few symptomatic tomato plants [138,148]. CEVd was also detected in Corsica [148] with restricted distribution. In France and in many European countries, PSTVd infections were found in several ornamentals: angel trumpets, chrysanthemum, petunia, cape gooseberry, marmalade bush, trailing petunia, Paraguay nightshade, climbing nightshade, Jerusalem cherry, and thorn apple (jimson weed) [166]. Measures taken in the context of PSTVd quarantine and certification led to the eradication of this viroid from France [153,154].

GYSVd-1 and HSVd (very often in association) are the most common and widespread grapevine viroids in France [57]. Other grapevine viroids detected were GHVd [72] and GYSVd-2 [155]. TASVd was found, mainly in symptomless ornamentals (angel trumpets, climbing nightshade, Paraguay nightshade, night blooming jessamine, marmalade bush) but also in tomato [166]. The situation in 2013 evaluated by EPPO [167] was that TASVd was absent, as in July 2013, when it was tested in garden centers located in different regions (Champagne-Ardenne, Pays-de-la-Loire, Picardie), and all asymptomatic but infected plants of angel trumpets, climbing nightshade, and tomato were destroyed. CLVd was detected in tomato [67], and HLVd in hop was widely detected (up to 90–100% of the tested hop germplasm) in France [57].

### 6.8. Greece

ADFVd was detected in fig, in 49% of the fig tree collection of the Agricultural University of Athens and in 6 out of 31 fig trees from other regions of the country [12,13]. ASBVd has few occurrences only in avocado [29]. ASSVd reached an incidence of 35% in cultivated and wild pears in Greece and Italy [20].

**Figure 3:**
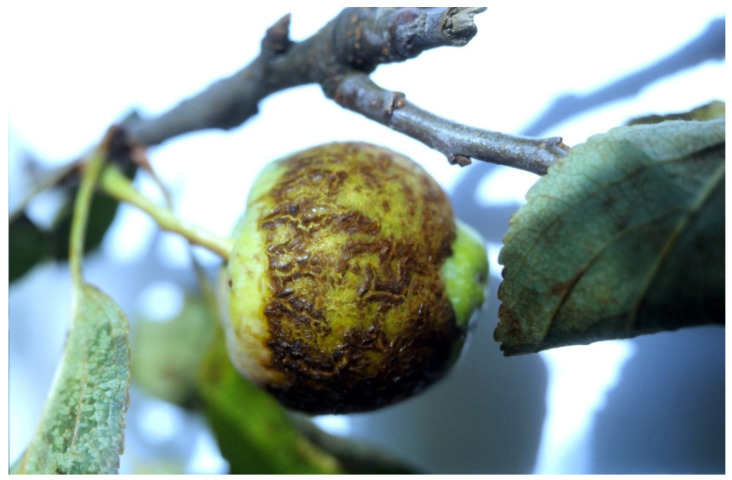
Apple scar skin viroid symptoms on an immature apple fruit from Olympia area of Peloponnesus, Greece.

ASSVd was also detected in sweet cherry [23]. In a survey of 400 Rosaceous samples tested by TPH, 117 were found ASSVd-positive (29.3%) [21]. Figure 3 shows symptoms of ASSVd on a Greek apple from Peloponnesus.

GYSVd was detected in symptomatic grapevines with asteroid mosaic symptoms along with HSVd [168]. During 2017–2018, surveys on grapevine, collected from symptomatic and symptomless vines from various cultivars in Heraklion (Crete) and Attica, led to verification of the presence of AGVd in 13 out of 298 samples tested by multiplex RT-PCR. The subsequent tests also revealed the presence of grapevine rupestris stem pitting-associated virus (GRSPaV), grapevine leafroll-associated virus-3 (GLRaV-3), GYSVd-1, GYSVd-2, and HSVd. Grapevine fanleaf virus, GLRaV-3, and GYSVd-2 were also detected in symptomatic AGVd-positive plants showing bright yellow and chlorotic discolorations, not allowing for the observed symptoms to be directly correlated with the AGVd infection [25,79]. In another case, GYSVd-1 and HSVd together with a badnavirus were identified by NGS in a Greek vineyard showing leaf discoloration symptoms [169].

In 2017, a symptomatic Cretan vine was subjected to HTS. Ten of the generated sequences were of viral/viroid origin and showed co-infection by GLRaV-3, GRSPaV, GYSVd-1, and HSVd. Moreover, a partial 281 nt sequence of GHVd was retrieved from the GenBank. GHVd is poorly characterized, and its relevant literature is very limited [71,72]. Fifty-one other randomly selected samples from commercial vineyards were screened for the presence of GHVd, and the viroid was detected in one vine cv. Soultanina by RT-PCR [73].

In surveys conducted before 2020, CEVd and HSVd were detected in symptomatic bergamot orange in 2002 [170]; CEVd and HSVd were also detected in the Epirus district in 43 and 73 out of 123 citrus samples collected (lemon, sweet orange, sour orange, mandarin, and Clementine) [171]. In a survey inspecting the Greek national citrus germplasm foundation collection (PAS), 47% of the budwood tree source samples were positive for one or more viroids. CEVd and HSVd were detected along with CBCVd, CBLVd, and CDVd. All samples tested negative for CVd-V, CVd-VI, and the CBLVd variant CVd-I-LSS [172,173]. A large-scale epidemiological study of citrus viroids in five districts, 38 locations, and 145 fields in Greece, based on the analysis of 3005 samples collected from 29 cultivars of six citrus species, took place during recent years. Five citrus viroids reported so far in the Mediterranean, namely CEVd, HSVd, CDVd, and CBCVd show a high frequency and wide distribution in all areas and in almost all hosts, whereas CBLVd is restricted to the island of Crete. Mixed infections were found in all districts where viroids are widespread [39], confirming the observation that CEVd may form complexes with other viroids in citrus trees [174]. In contrast, another large-scale survey of 1,738 samples that was recently conducted in two important citrus growing areas in Greece (Chania–Crete, and Argolis–Peloponnesus), for the identification of five of the citrus viroids, showed a widespread occurrence of HSVd and CDVd in six citrus hosts and almost in all different orchards, but lower infection rates of CEVd and CBCVd, whereas CBLVd had only few occurrences in specific hosts. Differences in the epidemiology appeared also between areas, hosts, and different varieties within hosts. In another survey that was conducted in Heraklion, 394 samples from five hosts (sweet orange, lemon, mandarin, lime, grapefruit) were collected mostly from asymptomatic trees and, in some cases, showing symptoms like leaf yellowing, stunting, and shoot bark cracking. These samples were tested by RT-PCR, and results revealed the incidence of all five viroids in the aforementioned citrus species, whereas the viroid distribution depended on specific host cultivar and variety. HSVd and CDVd showed the highest infection rates (65% and 45%) in all surveyed areas and citrus hosts, followed by CBCVd and CEVd with lower infection rates (21–35%), whereas CBLVd was detected only in sweet orange and mandarins [175]. These surveys showed that four of the five known citrus viroids in the Mediterranean are widespread in the country, regardless of the citrus material (i.e., mother trees, nursery plants, orchard trees, variety, or species), whereas CBLVd was restricted in orange, lemon, mandarin, and blood orange in Crete, as well as in citrus mother trees in Poros [39,148,159,160,161,171,172,173,176].

The general incidence of HSVd in Greece is high, that is, the viroid was detected in citrus, plum, peach, apricot, cherry plum, Japanese plum, apple, wild apple, grapevine, and fig, with an incidence rate of 38.9% (in a total of 232 from 596 samples tested by TPH), 39% in pome fruits, and ~50% in stone fruits [21,90,168,177]. According to earlier reports [57,89,149], HSVd was detected in apricot in Greece at a rate of 5%, yet, in a 2006–2009 survey, 124 out of 247 Greek apricot trees (~50%) were found HSVd-positive by tissue print hybridization [21].

PBCVd was detected in Greece [19], and, in a survey conducted by TPH, 112 out of 374 (30%) wild and domesticated pome fruit samples were PBCVd-infected. The local wide host range of PBCVd includes five pome fruit species (two wild species, wild pear, and hawthorn) [21].

PLMVd and HSVd are ones of the most widespread viroids in Mediterranean fruit trees, and most likely, of Mediterranean origin [19,178,179]. PLMVd was first found in Greece in 1998, in 37% of the tested pear (Greece and Italy) and Greek wild pear (*Pyrus amygdaliformis*) samples [19], and later in apricot and quince [22,124,180]. Surveys showed that over 75% peach, 46% plum, and 13% cherry samples from various regions of Greece were infected by PLMVd. Peach germplasm seemed to play an important role for the high incidence of the viroid in peach. The detectable viroid titer by real time RT-PCR in non-peach samples was, on average, 99.6% lower than in peach, giving a possible explanation to the difficulty of PLMVd detection in non-peach species [22]. PLMVd has restricted distribution in Greece, according to EPPO [148], yet it is considered one of the most important pathogens of stone fruits in the country.

During a survey of PSTVd in symptomless ornamentals in Greece, two out of 315 climbing nightshade and four out of 76 angel trumpets samples collected from 21 commercial nurseries around the country were found positive for PSTVd. In addition, EPPO reports that PSTVd is transient in Crete [153,154]. During the years 2015–2023, and in the context of phytosanitary control in Benaki Phytopathological Institute (BPI) in Athens Greece, PSTVd was detected in imported ornamental plants, namely, garden dahlia from Uganda and Kenya, petunia, trailing petunia, and geraniums from Spain, and in imported tomato, eggplant, and pepper seeds from USA, Thailand, Jordan, China, and India. In two cases, local pepper seed was found infected by PSTVd [181]. The aforementioned data suggest that PSTVd is both transient and established (under eradication) in Greece.

### 6.9. Israel

As shown below, in Israel, the first citrus viroid diseases were documented, and citrus viroids CBLVd and CBCVd from there have been characterized since the early 1990’s [40,182]. CBLVd is also recorded in avocado [183].

ADFVd has been characterized recently in pomegranate in Israel [11]. ASBVd was detected only in avocado, its natural host. The first record of avocado sunblotch disease in the Mediterranean was in Palestine in 1934 [184]. The disease has been under control (restricted distribution) in Israel since 1964 by using certified disease-tested avocado mother trees [30,148,185]. Incidence of CBCVd in citrus is recorded [148,186,187], the Israeli sequence (GenBank Acc.No. NC003539) being the CBCVd reference sequence. CBLVd was characterized from Etrog citron in 1991 [44]. The CBLVd variant CBLVd-225 (GenBank Acc.No. NC001651) is also the CBLVd reference sequence [45,160,188].

The incidence of HSVd in citrus in Palestine and Israel was both in trees with symptoms of cachexia (xyloporosis) disease (Figure 2) and citrus gummy bark disease, and as growing of traditional local citrus varieties and rootstocks declined over the decades, so did the incidence of these diseases [148,189]. CEVd variants are divided into two classes that differ in their pathogenicity on tomato as well as in the number of nucleotide changes as compared with CEVd type strains [45]. The incidence of CDVd is sporadic, and CDVd strains from Israel show extensive sequence diversity [48,161].

TASVd was found in greenhouse-grown tomato crops in Israel, where it causes severe symptoms and losses. The experimental host range of TASVd-Is is significantly different from that of the strain TASVd-Ivory Coast [54,190,191]. TCDVd is seed-transmitted in tomato [192], and this finding was confirmed in Israel [186]. PSTVd was detected in potato [153,154,187,193].

### 6.10. Italy

The disease caused by ADFVd was first observed in commercial orchards in southern Italy [6,7]. Furthermore, ADFVd was characterized in fig by NGS of small RNAs [9]. HSVd and other five citrus viroids, namely, CEVd (Sardinia, Sicily), CBCVd, CBLVd, CDVd, and CVd-V, were detected in citrus infected with CTV by NGS [148,159,160,161,194]. HSVd was also present in citrus in Sardinia [148]. CDVd was mainly found in Sicily [161,195]. The incidence of HSVd in apricot was 37.2% [57,196]. HSVd was also reported on chickpea [141]. PLMVd incidence was 0.4–70% depending on the host; more than 70% of peach and nectarine cultivars were found infected with PLMVd, whereas the infection rate in commercial orchards was about 50% [113]. PLMVd was detected in few occurrences in plum (0.4%) [112] and apricot (2%) [196]. PLMVd has also been affecting clingstone peach germplasm [197], and it has been occasionally detected in sweet cherry [114]. According to EPPO, PLMVd now has restricted distribution in this country [148].

ASSVd was detected reaching an incidence of 35% in cultivated pears [20]. PBCVd was also detected in Italian pear [19,197]. GYSVd-1 and HSVd (very often in association) are the most common and widespread grapevine viroids [26,57]. GYSVd-2 and CEVd were reported only sporadically [155,198], and AGVd was also reported [26]. GLVd was detected by NGS analysis in grapevine cvs. Adissi, Rkatsiteli, and Katta Kourgan, originally from Armenia, Georgia, and Uzbekistan, respectively, and in one plant from *Vitis riparia* (cv. Gloire de Montpellier), originally from North America [74]. CSVd is a quarantine pest in the EU since 2009, because it causes stunting, leaf chlorosis, and floral disorders on chrysanthemum and has been reported in several European countries. CSVd spread in Europe has been efficiently restrained so far, although several outbreaks have been recorded. It is currently present, with restricted distribution in mainland Italy and Sicily [145,148], in different hosts: chrysanthemum and marguerite daisy [165]. CSVd was detected in several symptomless cultivars of marguerite daisy [199].

The incidence of PSTVd, TASVd, and CEVd in several ornamental species was recorded in few occurrences, and sporadic PSTVd outbreaks in tomato have been also reported along with TASVd [60,132,145,153,166]. In a survey that was carried out between 2009 and 2010 [60], in a total of 111 symptomless plants collected from several Italian nurseries and gardens, 48 plants tested positive for pospiviroids infection by the viroids: PSTVd (25/35 climbing nightshade and 22/29 night blooming jessamine), TASVd (4/35 climbing nightshade), and CEVd (1/29 night blooming nightshade and 1/5 Paraguay nightshade). TASVd was identified for the first time in Italy in climbing nightshade plants, two of them co-infected with PSTVd. CEVd was identified for the first time inParaguay nightshade and night blooming jessamine, in the latter mixed with PSTVd. CLVd was detected in tomato [68], and IrVd-1 was detected in common purslane, featherhead amaranth, and cockscomb [102].

### 6.11. Lebanon

ADFVd was found in infected symptomatic apple trees [8,10]. The incidence of PLMVd infection in peach trees was about 34%; PLMVd was detected in 17/50 samples belonging to native and international peach varieties (Babcock, Chikhani, Springtime, Nectarose, Dixired) collected from Mount Lebanon, north Lebanon, and Békaa Valley [200]. PLMVd is present in stone fruit in a significant percentage, 34% [201]. HSVd incidence in apricot was 27.6% [57,89,92]. HSVd was found in 36/130 apricot samples all belonging to native apricot varieties (Baiadi, Sindiani, Dehabi, and Aajami) collected from the northern area of the Békaa Valley [200]. HSVd was also detected in symptomless mulberry trees with an infection rate of 10% [94]. Subsequently, HSVd was reported in fig at an infection rate of 13.3% [93].

CEVd and HSVd were found in 33.3% of citrus samples with 66.7% infection in Valencia orange; CEVd was detected in 50% of Washington navel orange [53]. Incidence of CEVd was also reported by the EPPO [148]. Incidence of CBCVd in citrus was reported by the EPPO [38,148,159]. CBLVd and CDVd were also detected in citrus budlings originating in Lebanon [38].

### 6.12. Libya

CEVd and HSVd were reported to co-infect citrus [46,145,148].

### 6.13. Malta

There is a reference of PBCVd infecting the local pear cv. Bambinella, which is of special interest for the country. In 113 asymptomatic trees grown near Rabat, 14 (~12%) tested positive for PBCVd [123]. There are also a few occurrences of PSTVd, first found in 2012 in five symptomless angel trumpets in the nursery of a garden center in Burmarrad (a locality in St. Paul’s Bay). All infected plants were destroyed [153,154].

### 6.14. Monaco

There have been no references on viroids found in this country.

### 6.15. Montenegro

HSVd was reported in peach and nectarine [105], and PLMVd in peach [105,202]. CEVd was reported in common verbena [59,148,166]. In 2011, 80 samples of symptomless ornamentals and weeds belonging to genus *Solanum*, as well as asymptomatic solanaceous and symptomatic sweet pepper and tomato plants, were taken from eight production places and greenhouses. These samples were tested by RT-PCR for pospiviroids with genus specific primers. An amplicon of an expected size (approximately 196 bp) obtained from one common verbena sample underwent direct sequencing, and sequence analysis revealed the presence of CEVd, which was confirmed by CEVd-specific RT-PCR. The infected common verbena plants were not destroyed, since CEVd is not listed as a quarantine organism in Montenegro; however, any possible spread of CEVd infection to tomato could devastate the production of this crop in the country [59]. Also, detection of PSTVd in ornamental plants (three asymptomatic samples of climbing nightshade and one of angel trumpets collected in the municipality of Kotor) was reported [153,154,203].

### 6.16. Morocco

HSVd incidence in apricot was 1.2–10.3% [57,89,204]. The viroid incidence in citrus was 10.8% [205,206,207,208]. During 2008–2018, 5390 samples from 100 commercial citrus orchards of different varieties were collected from the main citrus growing areas, such as the Gharb region, for laboratory analyses using imprint hybridization and RT-PCR assays with specific primers. Viroid incidence was 29–41.5%, regardless of citrus species and location. CEVd was detected in 11.3–18.2%, while HSVd was detected in up to 10.8% of the inspected samples. Since CEVd and HSVd can be transmitted by working tools, they were found to affect both old and young orchards in all the surveyed regions [148,208].

The incidence of PLMVd was reported [57,115,148,204,209], and Bouani et al. [204] reported an incidence rate of 7.3% in apricot, plum, and peach. Ouantar et al. [210] reported that in summer 2013, during a field survey for the presence of CBLVd in Gharb commercial orchards, 25 symptomless common Clementine samples were assayed by RT-PCR, and amplicons of the expected sizes were obtained from 15 of them (60%) [160,210].

### 6.17. The Palestinian Authority

Five citrus viroids were detected in the West Bank of The Palestinian Authority; three of them have been characterized, namely, CEVd, CDVd, and CBCVd [41,211]. In a total of 42 samples, the incidence of CDVd, CEVd, HSVd, CBLVd, and CBCVd was 35.7%, 23.8%, 9.5%, 7%, and 16.6%, respectively [41].

### 6.18. Slovenia

CBCVd has been detected in imported citrus fruits and in local hop since 2014 [148]. CBCVd has been found to cause severe stunting of hops; it is on the EPPO alert list with a restricted presence in the country (under eradication) [43]. According to Hagemann et al. [212], factors of spreading viroids internationally are fruit imports and inappropriate handling of fruit waste, such as deposition to agricultural lands. All five citrus viroids were detected in two surveys on imported citrus fruits and their RNA extracts, sap, and remains from grocery stores in Slovenia and in Germany. CBCVd, especially, is the causal agent of a severe form of hop stunt disease, and HSVd causes the homonymous disease in hops.The incidence of CSVd was reported in chrysanthemums in 2009–2010 [213] and in a few occurrences in 2017, yet currently, it is no longer present [148]. There is incidence of HSVd in a few occurrences in hops. It was first observed in hop gardens in 2007 in the Savinja valley and Koroška region, and now it is under eradication. This was also the first time that HSVd was detected in hops in Europe [148].

TCDVd was also reported in a few occurrences in symptomless petunia [139,148]. In 2010, 30 leaf samples, each out of five petunia species’ symptomless plants, were taken by phytosanitary inspectors from production sites in the country and were tested for the presence of PSTVd by real-time RT-PCR according to the EPPO protocol. A sample of cv. Surfinia Purple from a production site of the coastal region and another of cv. Surfinia Hot Pink 05 from a production site near Ljubljana were positive by real-time RT-PCR, by RT-PCR and by sequence analysis, to TCDVd. The infected petunia stocks were destroyed, as, despite TCDVd in petunia being symptomless, the infected plants could be a source of infection for tomato and potato, where it can cause severe damage, like that caused by infection with the closely related species of PSTVd [139]. TASVd was found in climbing nightshade plants in 2011 [137,166].

PSTVd was first detected in 2006 in symptomless ornamental *Solanaceae* (climbing nightshade, Paraguay nightshade, angel trumpets, petunia, and pepino). Eradication measures were taken [153,154]. About 25% of 398 samples collected in five years tested positive for PSTVd [135]. In another case, PSTVd was detected in 2011 in 10 young symptomless plants of cape gooseberry grown in a greenhouse. Plants were multiplied from an adult plant of unknown origin. RT-PCR and sequence analysis of four amplicons produced confirmed the presence of PSTVd. Cape gooseberry, as well as the aforementioned ornamentals, could potentially serve as a source of infection for tomato and potato, where PSTVd can cause severe losses [214]. CEVd was detected in symptomless climbing nightshade potted plants in 2010 [58,148,166]. Iresine viroid-1 was reported in common purslane [103].

### 6.19. Spain

ASBVd was detected originally in an avocado tree cv ‘Fuerte’. Sequence variation of ASBVd is common. Avocado production is mainly in the south-east part of Spain and the Canary Islands. PLMVd incidence in apricot, peach, and nectarine was 82% [215]. This viroid was found in 80–85% of foreign (North American) but not in local peach cultivars in the Valencia region [215], in 54% of peach germplasm in Zaragoza, and in about 50% of peach and nectarine (smooth peach) cultivars [216]. The viroid seems to have restricted distribution lately [148]. HSVd incidence in apricot was 80.9% in the surveyed trees [57,143,149,217]. HSVd prevalence in Spanish apricot is important [36].

Eradication of CSVd was reported by EPPO, as this is a quarantine pathogen in the EU [148]. In Spain, CSVd was reported mainly on chrysanthemums [165,218]. EPPO also reported the eradication of TCDVd, where it was found in eggplant seeds in 2019 [140,148]. PSTVd was also reported [153,154]. As in many European countries, PSTVd infections in Spain were found in several ornamentals since 1999 [145,166].

Incidence of CBCVd in citrus and trifoliate orange was reported [148,219]. In Spain, the first citrus viroid found in Europe, CEVd, was sporadically identified in Swedish turnip/rutabaga, carrot, broad bean, tomato, and eggplant, besides citrus [61,166,220]. The identification of CEVd is linked to the outbreak of the disease caused by CTV. The control of the tristeza disease was based on the substitution of the widespread sour orange rootstock by other species, most of which were sensitive to CEVd. Further investigations have shown the widespread occurrence of citrus viroids (CDVd, CBLVd, CBCVd, HSVd, CVd-V), different from CEVd, in all citrus growing areas [57,159,160,161]. CVd-V [62] was identified in Spain.

GYSVd-1 and HSVd (very often in association) are the most common and widespread grapevine viroids [57]. GYSVd-2 and CEVd were reported only sporadically in Spanish grapevine [198].

Recently, ADFVd has been characterized in pomegranate in Spain and Israel [11]. PBCVd was detected in the 1990s [121,122]. AHVd has recently been detected on loquat along with apple viruses [17]. HLVd was first identified in hop in Spain [80]. Subsequently, HLVd has been widely detected (up to 90–100% of the tested hop germplasm) [57]. CbVd-1 has also been found in several coleus cultivars [221].

### 6.20. Syria

Since the early 1980s, many virus and viroid diseases were investigated and reported on many fruit tree species, grapevine, nut trees, such as walnut, almond, other stone fruits, pome fruits, citrus, fig, grapevine, and olive. Assessment of the sanitary status of these fruit and nut trees and grapevine and viroid detection was carried out using the appropriate and available detection methods such as biological and molecular techniques [222]. PLMVd and HSVd were detected in stone fruits, such as peach at 40%, and apricot at 62.5% [57,95]. HSVd, CEVd, and CDVd were reported in citrus. Incidence of CEVd was also confirmed by molecular methods [223]. CDVd was detected in citrus budlings [38,223]. HSVd was detected in a sweet orange orchard in Tartous (sweet orange cvs. Jaffa and Valencia) and in another orchard in Lattakia (cv. Valencia). None of the infected trees exhibited conspicuous symptoms as they were all grafted onto sour orange rootstocks [148,223,224]. The citrus gummy bark disease, attributed to HSVd variants, was also reported in Syria [85]. HSVd was detected in fig, at an infection rate of 13.3% [96,222]. Finally, incidence of CBCVd or CBLVd on lemon, sweet orange, mandarin orange, grapefruit, and kumquat was reported [38,148,159,160].

### 6.21. Tunisia

Five citrus viroids (CEVd, HSVd, CDVd, CBCVd, and CBLVd), were reported by EPPO and CABI of their presence in the country [50,148,159,160,161,225]. In a field survey conducted in commercial orchards for estimating the prevalence of citrus viroids, 202 tree samples grafted on sour orange, including 75 trees showing cachexia symptoms, were examined by sequential polyacrylamide gel electrophoresis and molecular hybridization using viroid-specific probes. These tests revealed that all plants were infected with at least two viroids. Prevalence of CEVd, HSVd, and CDVd accounted for 70.3%, 72.3%, and 78.2% of the tested trees, respectively, while CBLVd and CBCVd were found in 28.2% and 3.0% of the same trees. The most frequent viroid combinations found were CEVd + HSVd + CDVd (34.7%) and HSVd + CDVd (22.3%), along with other less frequent combinations, such as CBLVd + HSVd + CDVd (12.9%), CEVd + CBLVd + HSVd (11.9%), and CEVd + CDVd (10.8%). CEVd is a prevalent viroid in Tunisian citrus [226] and its variants belong to class B (mild in tomato) [49]. CEVd was also found to infect fig in Tunisia [51]. Najar and Duran-Vila [226] reported that CBLVd affected, in mixed infection with other citrus viroids, the canopy volume and fruit quality of the Tunisian citrus cultivar Maltaise demi sanguine. CVd-V was also detected in most citrus varieties [63].

The incidence of HSVd in other fruit tree species was reported [148], in pear, peach, pomegranate, pistachio, and almond [97,98,227,228], while the first report of HSVd in fig trees was from Tunisia [51]. PLMVd is common in peach but not as common in pear [97]. A survey of fruit trees was carried out in 17 orchards in the northern and Sahel regions of Tunisia. Samples were collected in field trees of peach, pear, and almond that showed symptoms potentially caused by viroids (leaf mosaic in peach, blister canker in pear, necrotic leaves in almond). Twelve out of 37 peach trees tested were found infected with PLMVd, 2/73 of pear trees tested (2.7%) were infected with PBCVd, and HSVd was detected in 2/11 almond, 1/37 peach, and 7/72 pear trees tested. One pear tree infected with HSVd was also infected with PBCVd [97,227].

Recently, AHVd was reported in apple in Tunisia [229]. AGVd was detected from infected grapevines by RT-PCR and sequencing. The molecular variability of AGVd from cultivars Carignan and Syrah, was studied by restriction analyses and by sequencing [230]. GYSVd-1 was also found, and its genomic nucleotide sequence was determined [27]. PSTVd was detected in ornamental plants, but the viroid was apparently not reported by EPPO/CABI for this country.

TCDVd was detected in the ornamental mock orange; its nucleotide sequence shares identity with variants from Canada and The Netherlands. TASVd in tomato was also reported from Tunisia [136]. In May 2005, the Plant Protection Service in the Netherlands received two tomato plant specimens for diagnosis from a protected crop production facility of 2.5 ha near Kebili in Tunisia. Growth of the plants was reduced, and the leaves were chlorotic and brittle. Ripening of the fruits was delayed, and their storage life was reduced from 3 weeks to 1 week. The grower reported that initially, only 5% of plants showed symptoms; however, the number of symptomatic plants increased quickly to 100% because of increasing temperatures in the production facility. Tests comprising indexing on tomato, RT-PCR with universal pospiviroid and specific primers, and sequencing resulted in symptoms in tomato, amplicons of the expected size, and the expected nucleotide sequencing, respectively [136].

### 6.22. Turkey

ASBVd was detected only in avocado [32]. PLMVd was detected with an infection rate of 16–38% in peachand nectarine and 0–60% in apricot [117,118,149,231,232]. PLMVd was occasionally detected in the Malatya region of Turkey in persimmon, walnut, and sorghum [3,231]. ASSVd was detected in apple with an incidence of 46% in apple orchards in Eastern Anatolia [24].

Recently, ADFVd has been characterized in fig trees by RT-PCR and Sanger sequencing [14]. PBCVd was detected in 5.4% of pear and quince trees [8,127]. Incidence of CSVd in chrysanthemum was reported with an infection rate of about 1.3% [35,148]. PSTVd was detected in potato at the low incidence of 0.45–1.8% [35,153,154,193]. 

The history and properties of citrus gummy bark (CGB) disease were described by Önelge and Semancik [82]. An HSVd etiology for CGB disease of sweet orange is supported by the similarity of symptom expression to the cachexia (xyloporosis) disease of mandarins and tangelos caused by HSVd, as well as by the detection of variants thereof in CGB-infected Washington navel sweet orange and the Turkish orange cultivar “Dortyol” [233]. In addition, another HSVd-induced disease, citrus cachexia is also widespread in Turkish mandarin orchards [234]. Regarding fruit trees other than citrus, HSVd was detected in 14–20% of plum, 57–61% of peach, and 66–86% of apricot [57]. According to other surveys, the incidence of HSVd in apricot, plum, and peach trees was 0.1%, 0%, and 0%, respectively [117,149], and in another report, the incidence on apricot was found to be 2% [89]. HSVd infection of grapevine was about 6%, and the viroid was also found in grapevine rootstocks [77], as well as in pomegranate [99] and pistachio [101].

GYSVd-1, GYSVd-2, and CEVd were reported only sporadically in grapevine [77,155], as the infection rates for GYSVd-1 and GYSVd-2 in the Eastern Mediterranean Region of Turkey were 12.5% and 2.17%, respectively [77,78], whereas it was very low (0.09%) in Eastern Anatolia for the two viroids [235,236]. The two viroids also infect grapevine rootstocks [77]. Önder et al. [237] have used NGS technologies to identify viruses and viroids from a grapevine variety Trakya (Thrace) İlkeren showing severe mosaic symptoms in the Bursa province of Turkey in 2015. In final genome mapping results, among other grapevine viruses, a reference genome was recovered with indicated percentage of 100% for GYSVd-1. Also, AGVd has been reported in western Turkey vineyards [28].

CEVd usually forms complexes with other viroids in citrus trees [174]. In Turkey, these complexes consist of CEVd, CBLVd, HSVd, CDVd, and CBCVd [148,238]. CVd-V was also detected in sweet orange trees [64]. Two citrus viroids, CBLVd and CBCVd, were identified from citrus-gummy-bark-diseased sweet orange trees [148,159,160,183]. Dwarfed “Meyer” lemon or “Meyer lemon” on sour orange rootstock infected with CDVd and CBCVd was reported [42,161].

## 7. Origin, Evolution, and Spread of Viroids in the Mediterranean Basin

In 1989, it was proposed by Diener that the small size and circularity of viroids and certain viroid-like satellite RNAs may be “living fossils” from a pre-cellular RNA world [239]. Consistent with such a hypothesis, initial phylogenetic analyses indicated a monophyletic origin for viroids, selected plant satellite RNAs, and the viroid-like domain of human hepatitis delta virus RNA [240,241].

In this scenario, emergence of chloroplastic viroids (family *Avsunviroidae*) preceded that of nuclear viroids (family *Pospiviroidae*) [241]. A polyphyletic viroid origin, however, cannot be ruled out, particularly considering that the avocado sunblotch viroid is A+U rich in contrast to other viroids [4]. This RNA world hypothesis is still widely accepted as it gained additional supporting evidence that included the identification of other self-cleaving viroids and viroid-like satellite RNAs and the finding that in vitro-selected ribozymes can catalyze RNA synthesis [241]. In 2023, however, the outlines of an alternative hypothesis began to emerge. Two independent metagenomic analyses of environmental samples indicate that fungi provide an evolutionary hub for RNA viruses and viroid-like elements [242,243]. Furthermore, evidence has been presented for the transmission of the apple scar skin viroid from plant to plant-associated fungi under natural conditions [244]. The term “mycoviroids or fungal viroids” has been introduced to denote these viroids [245,246].

Viroid populations infecting a single host assume the features of quasispecies which are composed of closely related sequence variants. When inoculating a host with single infectious variants, the resulting progeny of members of the family *Pospiviroidae* and *Avsunviroidae* are heterogeneous, with the heterogeneity being higher in the latter. The host enzymes involved in viroid replication are presumed as the major cause for the sequence variability observed in viroids. There is a wide range of heterogeneity among the same viroid quasispecies in the same or different hosts around the Mediterranean. For instance, viroid variants like those of ASSVd in pome fruit and sweet cherry, PLMVd in peach, and PBCVd in Greece show less heterogeneity among Mediterranean countries, namely, Greece, Turkey, and Bosnia, than the heterogeneity among different hosts in the same country (see below). By contrast, genetically stable viroid populations were recorded, such as AGVd in grapevine, CEVd in various hosts, and the Italian population of CDVd in trifoliate orange, Troyer citrange, Etrog citron, and Navelina sweet orange; the latter preserved viroid variants similar to an original CDVd inoculum even after 25 years. However, CDVd variants in other hosts, such as Interdonato lemon and volkamer lemon, were completely different from initial inocula in the inoculated sources, shedding light on the role of the host on the genetic variability of CDVd [195].

### 7.1. ADFVd

The full genome (310 nt) of a Greek ADFVd variant from a fig tree cv. “Mavri Difori” showed 96.3% to 96.7% nt identity to other homologous ADFVd sequences from fig trees deposited in GenBank [12]. Turkish ADFVd fig isolates were more similar to Italian ones from fig and Spanish ADFVd isolates from pomegranate.

### 7.2. AHVd

AHVd apple isolates MH049330 and MH049331, MH049332 from Italy and Spain, respectively, showed 86.2 to 93.9% identity with the AHVd reference sequences KR605506 from China and isolate Pacific Gala (MF402929) from Canada.

### 7.3. AGVd

Sanger sequencing revealed the full AGVd genome for two AGVd isolates from Crete, Greece, AGVd S122 (369 nt; GenBank Accession No. MT227864) and AGVd Her75 (370 nt, MT227865). One of cv. ‘Mandilaria’ [VIVC 7300] from Heraklion) gave 29 AGVd sequences that shared 100% identity [25].

The molecular variability of Tunisian AGVd from cultivar Carignan and HTS of a pooled sample from four grapevines (three of cv. ‘Soultanina’ [VIVC 12, 051] and Syrah) was studied by restriction analyses and by sequencing. Sequence variability was not detected in any specific domain or region of the genome [229].

### 7.4. ASBVd

Two different ASBVd variants were identified from a symptomatic fruit originating from Chania, Crete, Greece, AvoCr0-A and B (Acc.No. LT966069 and LT966070, respectively). Nucleotide analysis results showed that the Greek variants of ASBVd are 95–99% identical to the already characterized isolates of ASBVd [29].

The Spanish ASBVd isolate from an avocado tree cv. ‘Fuerte’ showed heterogeneity in the two end loops of the molecule compared to an Australian isolate, but not in its central part, which harbors the ribozyme self-processing of the viroid plus and minus RNAs. The high sequence identity (98%) of these two isolates of such distant geographic localizations suggests their common origin [31], maybe due to imported and contaminated propagation material [31].

### 7.5. ASSVd

Forty-two complete ASSVd sequences from pome fruits and sweet cherry in Greece shared very high identity (99–100%) with Asian sequences from apple and significant variation among themselves (16%), equal to the variation within Chinese ASSVd sequences. Three ASSVd variants from apple (Pella), wild apple (Pella), and pear (Achaia) were found identical with each other and identical with another group of three apple variants from China [21]. It is possible that some ASSVd variants of the eastern Mediterranean originated from central Asia, where apple species came from.

### 7.6. CBCVd

There is a risk of introducing CBCVd, HSVd, and other viroids into hop growing regions via imported fruits, as CBCVd and HSVd sequence variants from imported fruits were almost identical to variants confirmed in hop [212].

CBCVd, collected from citrus-gummy-bark-diseased sweet orange trees in Turkey, was RT-PCR-amplified and directly sequenced. CBCVd sequence consisted of 285 nt. The obtained nucleotide sequence was comparable with the respective type strain previously sequenced from grapefruit in Israel [148,159,183].

### 7.7. CBLVd

CBLVd sequences of 327 bp from Moroccan citrus were deposited in GenBank under Accession Numbers MH200818, MH200819, and MH200820, and nucleotide blast analysis showed 99% sequence identity with CBLVd Japanese isolate P2 (AB006735), while phylogenetic analysis showed that the Moroccan CBLVd isolates were clustered in the same group [160,210].

A CBLVd isolate collected from citrus-gummy-bark-diseased sweet orange trees in Turkey was RT-PCR-amplified and directly sequenced. CBLVd sequence consisted of 317 nt. The obtained nucleotide sequence was comparable with the respective type strain previously sequenced from avocado in Israel [148,160,183].

### 7.8. CEVd

CEVd variants in Israel are divided into two classes that differ in their pathogenicity in tomato, as well as in the number of nucleotide changes as compared with CEVd type strains [45].

Cypriot CEVd isolates shared 97–100% identity to exocortis isolates from neighboring countries in Africa and Asia, suggesting multiple introductions through contaminated budwood [56]. RT-PCR amplification and sequence analysis of three Tunisian CEVd isolates showed 99% identity with each other and ~99% identity with those from Iran, Greece, and Syria [225].

An amplicon of an expected size (approximately 196 bp) obtained from a common verbena sample in Montenegro underwent direct sequencing, and sequence analysis revealed the presence of CEVd, which was confirmed by CEVd-specific RT-PCR. The CEVd sequence was deposited in GenBank (Accession No. JN872140) and had 99% identity with two other CEVd sequences from common verbena [59]. Italian CEVd sequences from Paraguay nightshade and night blooming jessamine were very homologous to each other (97 to 100% identity), showing few mutations, all located in the V domain of the pospiviroids [60].

### 7.9. CDVd

CDVd strains from Israel showed extensive sequence diversity which is attributed to point mutation, RNA recombination, and host characteristics like health status and variety [48,161].

The Italian population of CDVd was genetically stable in certain hosts (trifoliate orange, Troyer citrange, Etrog citron, Navelina sweet orange), but not in others (Interdonato lemon and Volkamer lemon) [195].

Tunisian CDVd isolates were 100% identical and shared >96% identity with other isolates from Brazil, Cyprus, Greece, Uruguay, Israel, and Spain [225]. In another study, the natural variability of CDVd was also reported [49].

### 7.10. CVd-V

CVd-V [62] was identified in Spain, and its sequence was deposited at the GenBank under the Accession Number NC010165.

The Tunisian CVd-V variants showed 80–91% identity with other known variants; two main CVd-V groups were identified [63].

The Turkish CVd-V isolate shared 96% and 98% identity with two different CVd-V reference isolates deposited in GenBank [64].

### 7.11. CSVd

French CSVd sequence of 355-nt (GenBank No. JF938538) shared 99% identity with CSVd isolates from chrysanthemum from Korea and Germany [164].

Italian CSVd isolates disclosed viroid RNA populations with a prevalent size of 354 nt and sequences 98–100% identical to those of other CSVd variants from chrysanthemum and marguerite daisy [199].

### 7.12. CbVd-1, CbVd-3

Croatian sequences from different coleus cultivars showed different phylogenetic grouping of these viroids, suggesting distinct infection sources, probably introduced in infected seeds [65,66].

### 7.13. GHVd

Greek grapevine PCR samples were subjected to HTS, and the sequences were deposited in GenBank under Accession Numbers MK791515 and MK7911516. Nucleotide analysis of the Greek full-length sequences of GHVd revealed 98.93% identity among themselves and 98.40 to 99.73% identity with the corresponding sequences of the deposited Italian (KR736334) and French (MF093720 and MF093719) isolates [73].

### 7.14. HSVd

Regarding HSVd variants from pome and stone fruit trees in Greece, eight of the 21 HSVd variants were found to belong to the HSVd-P group (dapple plum and peach fruit), while four other variants were grouped in the HSVd-H cluster (hop stunt). From variants belonging to these different clusters, two were found to coexist in a wild apple tree and the other two in two neighboring apple trees in an orchard. Three others, formerly characterized variants from Greek apricot [179], were clustered in the group HSVd P-H/C3, which was derived from recombination events between the previous two clusters. These findings are indications of such a possible event in Greece [21].

Sequencing analysis in Cypriot citrus showed that all HSVd sequences shared 100% nucleotide identity and were closely related to other variants from the Mediterranean Basin [56]. An HSVd variant from Egyptian citrus was associated with gumming and stem pitting in volkamer lemon rootstock, as it shared 100% identity with HSVd variants, such as CVd-IIc or Ca-905 (Cachexia-905 HSVd variant) [86]. Loconsole et al. [88] used real-time RT-PCR and HRM analysis to differentiate HSVd citrus variants from Mediterranean countries. HSVd variants from pome and stone fruit trees in Greece belong to three phylogenetic groups: HSVd-P group (dapple plum and peach fruit), HSVd-H (hop stunt), and group HSVd P-H/C3, which derives from recombination events between the previous two [21]. Nucleotide sequence analyses of cloned HSVd amplified products from Tunisian almond, peach, pomegranate, and pear revealed sequences similar to variants from other isolates previously characterized; some of them shared 99% to 100% identity with the HSVd from dapple plum fruit (GenBank Accession No. AY460202) [97,98]. Lebanese HSVd mulberry isolates shared 95–96% identity with other known HSVd isolates and clustered with HSVd-citrus, regardless of their geographical origin [94].

### 7.15. PLMVd

Sixty-six different Greek PLMVd stone fruit variants were classified together in group III [22], according to Ambrós classification [180], where all Turkish PLMVd isolates from peach belong to [232]. However, PLMVd detected in Turkish persimmon and in sorghum contained eight new sequence variants. Multiple alignment and phylogenetic analyses revealed that these variants clustered only with PLMVd-walnut isolates previously identified from Turkey, and their similarity with the PLMVd isolates detected in different fruit crops in the world was from 96.71 to 99.11% [3]. More than 100 new PLMVd variants were identified in Tunisian peach, pear, and almond trees and clustered into groups and subgroups [116]. Nucleotide sequence analyses of cloned PLMVd amplified products from Tunisia revealed sequences similar to variants from other isolates previously characterized for each viroid. PLMVd from Tunisian peach shared 85% to 96% identity with the PC-C32 Italian isolate of PLMVd from peach (GenBank Accession No. AJ550905) [116].

### 7.16. PBCVd

PBCVd Bosnian variants infecting a local pear variety have five new polymorphic positions in their genome, two of which are shared by all the sequenced Bosnian variants [120]. PCBVd Bosnian sequences share ~99% identity with Turkish (HM233936) and Chinese (KT183373) sequences from pear.

Fourteen of the 16 complete Greek PBCVd sequences have little homology with Australian sequences from pear (4-DQ198084, 35-DQ186640, 57-DQ186641), Japanese pear, and quince (565-DQ146342, 569-DQ146343), and the other two Greek PBCVd sequences have great homology (97–98%) with Bosnian and Australian sequences from pear (PB-BR; EF530210, 41-AY508474). At the same time, Greek sequences show significant variation among themselves (16%); this is double the variation of the rest, non-Greek sequences. Greek sequences are clustered in two groups, and most of them have slightly branched secondary structures [21].

Kyriakopoulou and her colleagues [19] suggested that PBCVd, PLMVd, and ASSVd may have originated in the Mediterranean in their host plant wild pear, *Pyrus amygdaliformis* (syn. *Pyrus spinosa*). This wild pear is native to the northern Mediterranean Region [247].

Nucleotide sequence analyses of cloned PBCVd amplified products from Tunisian pear revealed sequences 99% identical with the P47A isolate variant nine from Spanish pear (GenBank Accession No. Y18043) [227].

### 7.17. PSTVd

PSTVd Mediterranean isolates from potato and ornamental solanaceous plants seem to be linked, directly or indirectly, to Dutch, German, and American variants of the viroid. Sequencing of the four Greek PSTVd-positive PCR products showed that all angel trumpets isolates (GU481090) were identical to PSTVd isolate 3077740 from Dutch angel trumpets (EF192394). One of the climbing nightshade isolates was identical to PSTVd isolate 3373056 from Dutch climbing nightshade (EF192393). The other climbing nightshade isolate (GU481092) was 99% identical to an intermediate strain of PSTVd from American potato (AY937179). The four positive samples of angel trumpets (GenBank Accession No. GU481090) were collected from a single nursery in Attica, central Greece, and they originated from Italy. One climbing nightshade isolate (GU481091) was collected from a nursery in Messinia, Peloponnese, and classified as of domestic origin, while the origin of the second climbing nightshade isolate (GU481092) collected from a nursery in northern Greece is undetermined [134].

PSTVd Italian isolates showed two different patterns. Those from climbing nightshade were almost identical among themselves (99–100% identity), conserving the informative mutation sites identified in the Italian isolates and described in [132]. Those from night blooming jessamine were homologous from 94 to 100% among themselves, but they did not show the characteristic informational site; instead, they were more like the PSTVd reference sequence [60].

Analysis of the 96 complete PSTVd master sequences in Slovenia from symptomless ornamental *Solanaceae* showed that most of them were identical to the sequence variant of PSTVd found in Paraguay nightshade in Italy (EF459700). Phylogenetic analyses of Slovenian sequence variants and sequence variants from GenBank showed two clearly separated clusters with high variability within each cluster. Although most of the sequence variants from the same ornamental host tended to group together, sequence variants from the same hosts also showed high variability. On the other hand, different hosts were found to be infected by the same sequence variant [135].

The full viroid sequence of PSTVd in Slovenian cape gooseberry had 358 nucleotides, and it was deposited in the NCBI GenBank under the Accession No. JN543964. This isolate was 100% identical to the cape gooseberry isolate from Germany (GenBank Accession No. EU862231) and the tomato isolate from New Zealand (GenBank Accession No. AF369530) [214].

The Turkish PSTVd potato isolate had the highest sequence identity (99%) with an Italian one [131].

### 7.18. TASVd

The Israeli isolate of the viroid (TASVd-Is) shared 92%, 99%, and 87% sequence identity with the isolates from Ivory Coast, Indonesia, and Germany (TASVd-S), respectively [54].

The Italian TASVd sequences obtained from at least eight climbing nightshade clones were very homogeneous among themselves (99% to 100% identity) but less homogeneous (91 to 99% identity) with other sequences deposited in GenBank [60].

Sequences obtained from tomato grown in Tunisia showed the highest identity to the four isolates of TASVd in the NCBI GenBank and to tomato isolates of TASVd from Indonesia and Israel [136].

### 7.19. TCDVd

Both Slovenian TCDVd isolates from petunia consisted of 360 nucleotides and showed 98% identity with other petunia sequences (GQ396664, EF582392, EF582393, and DQ859013) and 100% identity to an isolate from tomato (AF162131) [139].

In addition to viroids spreading by plant pathogenic fungi [244], viroids may also spread via vegetative propagules, mechanical damage, seed, pollen, or biological vectors [245]. Vegetative propagation is the most prevalent mode of spread at the global, national, and local level, while further dissemination can readily occur by mechanical transmission through crop handling with viroid-contaminated hands or pruning and harvesting tools [245]. Additional factors of spreading viroids internationally are fruit imports and inappropriate handling of fruit waste, such as deposition to agricultural lands [212]. Biological vectors shown to transmit viroids include certain insects, parasitic plants, and goats [245]. Viroids may persist in dried infected tissue for many years as shown by the detection and identification of ASSVd by RT-PCR in a 10-year-old air dried apple tree twig with no disease symptoms (A. Hadidi, unpublished) and identification of PLMVd by RT-PCR in 50-year-old herbarium peach leaves showing symptoms of peach calico disease [248].

## 8. Conclusions

The Mediterranean Basin has served as a basic intercultural communication and transport hot spot in human history. It is possible that this hot spot was and still is an important hub for viroid transmission and evolution. The higher incidences of a wide range of viroids in countries surrounding the Adriatic and Ionian seas suggest viroids may have spread from this region of Europe to other neighboring continents.

Viroid information reported in Mediterranean countries is important for national and international plant protection organizations to control viroid diseases. A case in point is the citrus bark cracking viroid in Slovenia, where the viroid was transmitted from one host species (citrus) to another (hop), causing significant damage to the new host (severe stunting). Subsequently, the disease was controlled. In the Mediterranean citriculture, citrus cachexia (xyloporosis) and citrus gummy bark, caused by variants of HSVd, and citrus exocortis, caused by CEVd, pose a threat to the susceptible CTV-tolerant trifoliate rootstocks used to replace the usual citrus rootstock (sour orange) which is tolerant to these diseases. Many of the Mediterranean countries apply modern methods to detect, characterize, restrain, and eliminate viroids and the diseases they cause in various hosts of important economic significance such as citrus, grapevine, and other perennial and herbaceous crops. It is noteworthy that some viroids have not been detected in the Mediterranean, either due to the lack of respective host cultures such as coconut palms or due to the effective quarantine and certification measures taken, such as for CSVd. Finally, it is worth mentioning that olive trees, which originated in the eastern Mediterranean Basin and may be one of the most important Mediterranean crops, have not been reported to harbor any viroid species.

## Figures and Tables

**Table 1:** Geographical distribution and host range of viroids in the Mediterranean Basin.

Viroid Acronym		Country	Host Range	Reference
ADFVd		Italy	apple, fig	[6,7,8,9]
		Lebanon	apple	[10]
		Israel, Spain	pomegranate	[11]
		Greece, Turkey	fig	[12,13,14]
AHVd		Italy, Spain	apple, loquat	[15,16,17]
ASSVd		Greece	apple, sweet cherry, pear, wild pear, wild apple	[18,19,20,21,22,23]
		
		Italy	pear	[19]
		Turkey	apple	[24]
AGVd		Greece, Italy, Tunisia, Turkey	grapevine	[25,26,27,28]
ASBVd		Greece, Israel, Spain, Turkey	avocado	[29,30,31,32]
CChMVd		France	chrysanthemum	[33]
CSVd		Egypt, Turkey	chrysanthemum	[34,35]
		Slovenia, Spain	mainly on chrysanthemum but also on other ornamentals	[36]
		France, Italy	chrysanthemum, marguerite daisy, common ragwort, common periwinkle, petunia, trailing petunia, climbing nightshade	[36]
CBCVd (CVd-IV)		Egypt, Greece, Israel, The Palestinian Authority, Turkey	citrus	[37,38,39,40,41,42]
		Slovenia	hop	[43]
CBLVd (CVd-I)		Egypt, Greece, Israel, Lebanon, The Palestinian Authority, Syria	citrus	[38,39,41,44,45,46]
CDVd (CVd-III)		Croatia, Egypt, Greece, Israel, Lebanon, The Palestinian Authority, Syria, Tunisia, Turkey	citrus	[37,38,39,41,42,46,47,48,49]
CEVd		Algeria, Egypt, Israel, Lebanon, Libya, Morocco, The Palestinian Authority, Syria, Tunisia, Turkey	citrus, tomato, fig	[38,41,45,46,49,50,51,52,53,54]
		Prevalent in Europe	citrus, climbing nightshade	[36,39,47,55,56,57,58]
		Montenegro	common verbena	[59]
		Italy	night blooming jessamine, Paraguay nightshade	[60]
		Spain	turnip, carrot, eggplant	[61]
		Italy, Spain, Turkey	grapevine	[36]
CVd-V		Spain, Tunisia, Turkey	citrus	[62,63,64]
CbVd-1		Croatia, Spain	spurflower	[36,65,66]
CbVd-3		Croatia	spurflower	[36,66]
CLVd		France, Italy	tomato	[36,67,68]
ELVd		Spain	eggplant	[36,69,70]
GHVd		France, Italy, Greece	grapevine	[71,72,73]
GLVd		Italy	grapevine	[15,74]
GYSVd-1		Albania, Croatia, Cyprus, France, Greece, Italy, Spain, Tunisia, Turkey	grapevine	[19,20,36,46,54,57,75,76,77]
GYSVd-2		Greece, Italy, Turkey	grapevine	[26,76,77,78,79]
HLVd		France, Spain	hop	[36,57,80]
HSVd		Countries of the Mediterranean Basin	citrus, grapevine	[36,46,54,57,81,82]
		Egypt	apple, pear, plum, peach, apricot, citrus, grapevine, mango	[54,83,84,85,86]
		Croatia, Bosnia and Herzegovina, Slovenia,	apricot, plum, grapevine	[47,87]
		Albania, Algeria, Cyprus, France, Libya, Italy, Israel, Spain	apricot, plum, peach, citrus, grapevine	[36,54,56,88,89]
		Greece	almond, wild almond, plum, sweet cherry, Japanese plum, peach, apricot, apple, wild apple, grapevine, citrus	[36,89,90,91]
		Lebanon	apricot, fig, mulberry, citrus, grapevine	[54,92,93,94]
		Syria	fig, citrus, apricot, grapevine	[54,88,95,96]
		Tunisia	pomegranate, citrus, fig, pistachio, almond, peach, pear, grapevine	[54,97,98]
		Turkey	pistachio, citrus, pomegranate, grapevine, apricot, plum	[54,88,89,99,100,101]
IrVd-1		Italy	featherhead amaranth, cockscomb	[36,102]
		Italy, Slovenia	common purslane	[36,103]
PLMVd		Widespread in Europe	peach	[36,57]
		
		Bosnia and Herzegovina, Croatia	peach	[87,104,105]
		Egypt	apple, apricot, mango, plum, pear, peach	[46,52,54]
		France, Algeria	apricot, sweet cherry, plum, peach	[106,107]
		Albania	apricot, plum, peach	[107]
		Greece	peach, wild pear, pear, hawthorn, plum, apricot, cherry, Japanese plum, quince	[19,22,36,106,107,108]
		Italy	apricot, cherry, Japanese plum, plum, Nanjing cherry, pear	[36,57,106,107,109,110,111,112,113,114]
		Lebanon, Morocco, Syria	apricot, sweet cherry, stone fruits	[46,54,95,106,107,115]
		Slovenia	plum	[107]
		Tunisia	almond, peach, pear	[54,97,106,107,116]
		Turkey	apricot, peach	[54,117,118]
PBCVd		Albania, Bosnia and Herzegovina, France, Spain, Italy, Malta	pear	[36,119,120,121,122,123]
		Greece	apple, pear, quince, wild pear, wild apple	[19,124,125,126]
		Tunisia, Turkey	pear, quince	[54,97,127]
PlVd-I		Israel	pomegranate	[11,128]
PSTVd		Egypt, Israel, Turkey	potato, tomato	[35,46,54,129,130,131]
		Italy	tomato	[36,132]
		Prevalent in Europe	ornamentals	[36,133,134,135]
TASVd		Israel, Tunisia	tomato	[36,46,54,136]
		Croatia, France, Italy, Slovenia	angel trumpets, climbing nightshade, Paraguay nightshade, night blooming jessamine, marmalade bush	[36,133,137]
		France, Israel, Italy	tomato	[36]
TCDVd		France, Israel, Tunisia	tomato	[36,54,138]
		Tunisia	mock orange	[54]
		Slovenia	petunia	[36,139]
		Spain	eggplant	[140]

## Dedication

This article is dedicated to the memory of the Mediterranean scientists who were experts in viroid research: Esmat K. Allam (Egypt), Martin Tabler (Greece), Jean Claude Desvignes (France), Luciano Giunchedi (Italy), Ricardo Flores (Spain), Mohamed Marrakchi (Tunisia), and Mona Gazel (Turkey). The article is also dedicated by the senior author to the memory of a faith fighter, St. Lucas Voyno Yasenetsky, who inspired her to conceive it and write it.
